# Combinatorial effects of multi-site stimulation on depression-related brain regions: clinical data analysis and predictive modeling

**DOI:** 10.3389/fpsyt.2026.1808486

**Published:** 2026-04-30

**Authors:** Atefeh Ghazavi, Koorosh Mirpour, Masoud Soroush, Eleonora Bartoli, Nisha Giridharan, Denise Oswalt, Nicole R Provenza, Anusha Allawala, Wayne Goodman, Sameer A. Sheth, Nader Pouratian

**Affiliations:** 1 Department of Neurological Surgery, University of Texas Southwestern, Dallas, TX, United States; 2 Department of Computer Science and Electrical Engineering, University of Maryland Baltimore County, Baltimore, MD, United States; 3 Department of Neurosurgery, Baylor College of Medicine, Houston, TX, United States; 4 Department of Neurosurgery, University of Pennsylvania, Philadelphia, PA, United States; 5 Department of Neurological Surgery, University of California San Francisco, San Francisco, CA, United States; 6 Department of Psychiatry, Baylor College of Medicine, Houston, TX, United States; 7 Department of Neurology, University of Texas Southwestern, Dallas, TX, United States

**Keywords:** additive interaction, deep brain stimulation, local field potential, multi-target stimulation, stereo-electroencephalography, treatment resistant depression

## Abstract

**Background:**

Despite growing evidence supporting deep brain stimulation (DBS) for treatment- resistant depression (TRD), how stimulation delivered across hemispheres or across multiple targets interact to shape large-scale network activity remains poorly characterized.

**Objective:**

Using a unique opportunity to simultaneously stimulate the subcallosal cingulate (SCC) and ventral capsule/ventral striatum (VC/VS) in subjects with TRD while recording neural activity across putative prefrontal networks underlying depression via intracranial electrodes, we investigated whether bilateral or multi-target stimulation has additive, synergistic/super-additive, or antagonistic/sub-additive effects on power modulation across depression-related brain networks.

**Methods:**

Four DBS leads, and ten stereo-electroencephalography (sEEG) leads were implanted in depression-related prefrontal brain regions in three subjects with TRD. Power modulation in response to unilateral and bilateral stimulation, as well as interaction classes of combinatorial stimulations, were evaluated across various combinations of frequency bands and region of interests (ROI) using marginal predictions from a linear mixed-effects model which were then used as input for machine learning classifiers to predict the additive interaction class of combinatorial stimulations.

**Results:**

Bilateral and multi-target stimulation produced additive or sub-additive interactions in most cases. A decision tree classifier identified ROI as the most important feature for predicting interaction class, followed by stimulation target and spectral frequency band.

## 1 Introduction

Major depressive disorder (MDD) affects approximately 4.4% of the world’s population (1), one third of whom do not respond to medications or psychotherapy and are categorized as having treatment resistant depression (TRD) (2). In the United States, this corresponds to approximately 2.8 million adults and $44 billion economic burden per year (3). Deep brain stimulation (DBS) has been actively investigated as a therapeutic option for TRD. Despite the initial promising results in open-label studies, double blind studies on the efficacy of DBS for TRD have not successfully demonstrated therapeutic benefit (3–6). Given the mixed outcomes, we have previously suggested that tailoring DBS to the individual’s depressive phenotype by employing multi-site stimulation targeting subcallosal cingulate (SCC) and ventral capsule/ventral striatum (VC/VS) and adjusting stimulation parameters may enhance treatment efficacy (7).

Beyond common parameters such as amplitude, pulse width, frequency, and contact used in single- site stimulation, combining stimulation sites across hemispheres and across targets may offer a unique strategy to improve DBS effectiveness. Tractography and functional imaging studies have demonstrated that SCC is structurally and functionally connected to a distributed network of cortical and subcortical regions such as medial frontal cortex, rostral and dorsal anterior cingulate cortex, pregenual, midcingulate, and posterior cingulate cortices, ventromedial and dorsomedial prefrontal cortex, nucleus accumbens, anterior thalamus, hypothalamus, amygdala, hippocampus, insula, brain stem, periaqueductal gray, and medial/central orbitofrontal cortices (8–12). In comparison, VC/VS connects dorsal prefrontal cortex, dorsal anterior cingulate cortex, orbitofrontal cortex, and ventromedial prefrontal cortex to thalamus, amygdala, hippocampus, hypothalamus, and brain stem (13). Given the common regions of connection, we hypothesized that multi-target neuromodulation of depression-related brain networks does not always result in additive interaction.

The present study aims to characterize the modulatory effects of different stimulation targets, individually and in combination, on local field potential (LFP) power across depression-related brain network regions of interest (ROI) and frequency bands. We assessed both the direct effects of stimulating individual targets and their combinatorial interactions to determine whether their effects were additive, super-additive or sub-additive. Here, we use the platform afforded to us by an ongoing trial of stereo-electroencephalography (sEEG-) guided DBS for TRD (NCT03437928) to assess the interaction of power modulation of the prefrontal depression networks. By leveraging linear mixed-effects (LME) modeling and bootstrap-based confidence intervals, we quantified the magnitude and significance of these interactions. This framework provides insight into potential synergistic or antagonistic relationship between stimulation of different targets, which is critical for gaining insights into interactions in modulatory effects of stimulation across hemispheres and across therapeutic targets in TRD and may provide insights into how combinatorial stimulation may be employed in other therapeutic strategies.

## 2 Materials and methods

### 2.1 Experimental paradigms

Neurophysiological data was collected from three participants (a 32-year-old woman, a 61-year-old man, and a 33-year-old woman) who were diagnosed with TRD and met inclusion criteria of severity, chronicity, and treatment refractoriness and provided informed consent according to Baylor College of Medicine IRB (H-43036) and University of Texas Southwestern IRB (STU2021-0724). They were enrolled in an ongoing FDA-approved NIH-funded clinical trial (IDE G180300, NIH grant UH3-NS103549).

We implanted 10 sEEG leads (PMT Corp., MN, USA), with five leads per hemisphere to record neural signals from the putative prefrontal depression-related network, including amygdala/hippocampus (Amy/HC), orbitofrontal cortex (OFC), dorsolateral prefrontal cortex (DLPFC), anterior cingulate cortex (ACC), temporal lobe (TL), and medial prefrontal cortex (mPFC) in conjunction with the implantation of bilateral externalized DBS leads targeting VC/VS and SCC (four total DBS leads). The participants underwent 10 days of intracranial recording to study the brain networks. At the end of this study period, the sEEG leads were explanted and DBS leads were connected to implanted pulse generators (IPG) for chronic stimulation.

#### 2.1.1 Electrical stimulation of SCC and VC/VS

Using four DBS leads (Vercise Cartesia directional leads and 8-ring leads, Boston Scientific Corp., CA, USA) targeting the bilateral SCC and VC/VS, we evaluated the effects of seven different combinations of stimulations on LFP power: (1) right SCC alone, (2) left SCC alone, (3) right VC/VS alone, (4) left VC/VS alone, (5) simultaneous bilateral SCC, (6) simultaneous bilateral VC/VS, and (7) simultaneous bilateral SCC and VC/VS.

The 8-ring lead contained eight ring contacts, each with a surface area of 6 *mm*^2^. The directional lead consisted of a dome-shape tip contact with a surface area of 6 *mm*^2^, six segmented contacts with individual surface areas of 1.5 *mm*^2^ (each covering 90 degrees of the lead’s circumference), and one ring contact with 6 *mm*^2^ surface area. The current return electrode for neural stimulation was a polyhesive electrode (Valleylab Polyhesive electrode, Medtronic Inc., MN, USA) placed on the participant’s leg.

**Figure 1:**
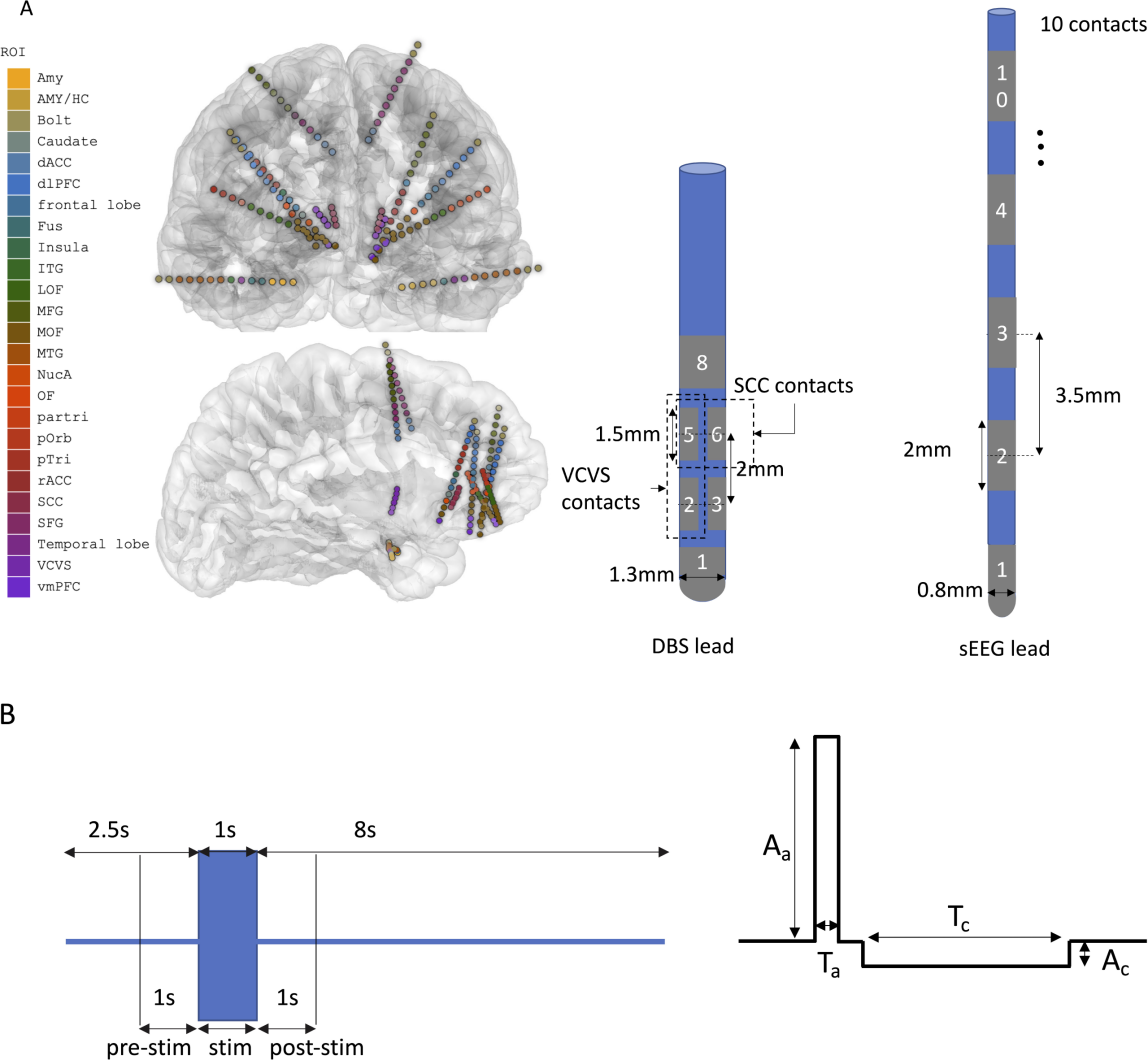
**(A)** Representative figure of frontal and medial views of the reconstructed cortical view and the DBS and sEEG contacts’ locations in the brain for participant 1 (left), DBS and sEEG leads with respective contact geometry (right). SCC stimulation was delivered to contacts 5,6, and 7 and VC/VS stimulation was delivered to contacts 2 and 5 of the DBS lead. **(B)** Stimulation protocol (left) and stimulation pulse (right) for subject 1. 1 second stimulation with 8 seconds interphase delay was delivered using a biphasic charge-balanced asymmetric anodal-first rectangular current pulse (Ac, Aa and Tc, Ta are the cathodal and anodal pulse amplitude and duration, respectively).\.

Stimulation parameters were based on a combination of commonly reported therapeutic settings in the literature (14) and additional methods described below. Biphasic charge-balanced rectangular current pulses were used to stimulate the target regions. 130 Hz monopolar stimulation with 90*µ*s and 180*µ*s pulse width was delivered in a random order, with 10 one-second repetitions of each stimulation combination. Stimulation parameters were further defined using three strategies, including based on 3D Illumina algorithm (15) (Illumina-guided), monopolar mapping of acute behavioral effects of stimulation (behavioral-guided), and stimulation-field modeling of monopolar activation that maximally engaged relevant structural brain networks based on MR diffusion tractography (16) (image-guided). Not all stimulation types (Illumina-guided, behavioral-guided, image-guided) were applied in every participant. The stimulation parameters used for each participant are summarized in the **Supplementary Table 1**. A representative figure of the reconstructed image of the contacts’ location in the brain, the DBS and sEEG electrodes and stimulation paradigm used for participant 1 is shown in **Figure 1**.

#### 2.1.2 Neurophysiological recordings

The neural data was recorded in a monopolar configuration at the rate 30,000 samples/second using NeuroPort data acquisition system (Blackrock Neurotech, UT, USA) with hardware Butterworth filters with cut off frequencies set at 0.3 Hz (high pass, order:1) and 7.5 kHz (lowpass, order: 3) and system line noise cancelation enabled. sEEG leads used for LFP recording consisted of 10 ring-shaped platinum contacts, each with a surface area of 5*mm*^2^ (**Figure 1A**).

### 2.2 Analyzing power modulation

For pre-processing, recorded voltages on each channel were bipolar re-referenced relative to the adjacent channel (provided both channels were located within the same region) and the data was decimated to 1 kHz. Each stimulation epoch consisted of one second of stimulation (stimulation period), one second before and after stimulation (pre-stimulation and post-stimulation periods). The continuous wavelet transform using bump wavelet was employed for power analysis and the power was averaged at four frequency bands (theta (4–8 Hz), alpha (8–12 Hz), beta (13–35 Hz), and gamma (36–50 Hz)) during pre-stimulation and post-stimulation periods. The averaged power during post-stimulation period was normalized to the averaged power during the pre-stimulation period for each frequency band. A window of 100 ms from the end of the pre-stimulation period and beginning of the post-stimulation period was excluded from the averaging.

Due to the large size of dataset, we used a deep learning approach to enable efficient outlier removal (17, 18). To exclude outliers from analysis, we manually labeled the spectrograms of one dataset that were saved as PNG images (5500 images) into spectrograms with and without outliers. Outlier spectrograms were identified based on the presence of wavelet edge effects at stimulation onset and offset. We then trained a convolutional neural binary classifier to label the rest of the datasets. We implemented this CNN model using the TensorFlow library to classify the images into normal and outliers. The input images were RGB with a resolution of 674 *×* 900 pixels. The dataset was split into train and test datasets (80% train, 20% test) and trained using Adam optimizer with categorical cross entropy as the loss function. The train dataset comprised of 4424 samples and the F1-score was used to assess the performance of the CNN classifier. Approximately 9.7% of the test data was identified as outlier. The machine learning model to classify the outliers could predict spectrograms that were considered “normal” and not outlier in the test data with 99% accuracy and F1-score of 0.99.

To facilitate analysis, anatomically distinct subregions identified by FreeSurfer were aggregated into larger ROI. These ROI included (1) ACC, consisting of anterior part of the cingulate gyrus and sulcus, mid-anterior portion of the cingulate gyrus and sulcus (2) mPFC, consisting of transverse frontal gyrus and sulcus (3) OFC, consisting of orbital gyrus, gyrus rectus, lateral orbital sulcus, inferior frontal gyrus, and suborbital sulcus (4) DLPFC, consisting of inferior frontal sulcus, superior frontal gyrus, superior frontal sulcus, middle frontal gyrus, and inferior frontal gyrus (5) Amy/HC, consisting of amygdala, hippocampus, and medial occipito-temporal gyrus (6) TL, consisting of superior temporal sulcus, lateral superior temporal gyrus, planum polare, middle temporal gyrus, occipital temporal medial sulcus and the lingual gyrus. We analyzed power modulation using an LME model to account for repeated measurements and nested data dependencies (19, 20). The model framework included: 1. fixed effects (stimulation target, ROI, and frequency band) and their two-way and three-way interactions to capture overall trend across key variables and their interactions. 2. random intercepts (recording channel nested in ROI, trial nested in dataset nested in subject) representing group-level variance (e.g., individual subjects, or recording channels). The averaged power during post-stimulation period (*P_post−stim_*) was normalized to the averaged power during the pre-stimulation period (*P_pre−stim_*) for each frequency band. The primary outcome was the relative change in power (Δ*P)* defined as (*P_post−stim_ − P_pre−stim_*)*/P_pre−stim_* for each stimulation target at each ROI and frequency band. We derived population-level predictions to evaluate differences between post-stimulation and pre-stimulation power. Statistical significance was assessed using bootstrap technique with 999 resamples to estimate the p-values (21) and the estimated p-values were corrected for multiple comparisons using false discover rate (FDR) method (22).

### 2.3 Additivity analysis

Because each interaction involves a specific subset of stimulation, the dataset was partitioned into three subsets, and three separate LME models were fit. 1. Interaction between right SCC and left SCC stimulation (rSCC, lSCC and bilateral SCC data were used), 2. Interaction between right VC/VS and left VC/VS stimulation (rVC/VS, lVC/VS and bilateral VC/VS data were used), 3. Interaction between bilateral SCC and bilateral VC/VS stimulation (bilateral SCC, bilateral VC/VS and simultaneous bilateral SCC and VC/VS data were used). For each subset, additive interaction was evaluated using the standard additivity formula (23), Interaction = Δ*P_AB_ −* (Δ*P_A_* + Δ*P_B_*), in which Δ*P_AB_* is the relative change in power due to simultaneous stimulation of target A and B, Δ*P_A_* is the relative change in power due to stimulation of target A, and Δ*P_B_* is the relative change in power due to stimulation of target B. To evaluate interactions between stimulation combinations, we used an LME model with stimulation target, ROI, and frequency band as the fixed effects, recording channel nested in ROI and trial nested in dataset nested in subject as the random variables, and Δ*P* as the response variable. Δ*P* was estimated using predicted responses from the LME model for all ROI-frequency band combinations. Bootstrap resampling (N = 999) was utilized to compute the median interaction and associated p-values. Multiple comparisons were addressed using FDR correction. For each ROI-frequency band combination, multi-target stimulation was considered super-additive when FDR-corrected p-values *<* 0.05 and median interaction (Δ*P_AB_ −* (Δ*P_A_* + Δ*P_B_*)) had the same sign as the expected additive effect (Δ*P_A_* + Δ*P_B_*), sub-additive when FDR-corrected p-values *<* 0.05 and median interaction had the opposite sign of the expected additive effect, and additive when FDR-corrected p-values *≥* 0.05.

### 2.4 Modeling additive interaction

We used decision tree classifier in machine learning to predict interaction class of a combinatorial stimulation and to determine the primary factors contributing to the additivity classification. Input features included: stimulation target, ROI, spectral frequency band, and pre-stimulation power of each ROI during stimulation of target 1 and stimulation of target 2, and the target variable was the interaction class with the following outcomes: additive, sub-additive, super-additive. The dataset was partitioned into training and test sets with 70% of the data allocated for training and 30% for testing.

## 3 Results

### 3.1 Modulated regions

**Figure 2:**
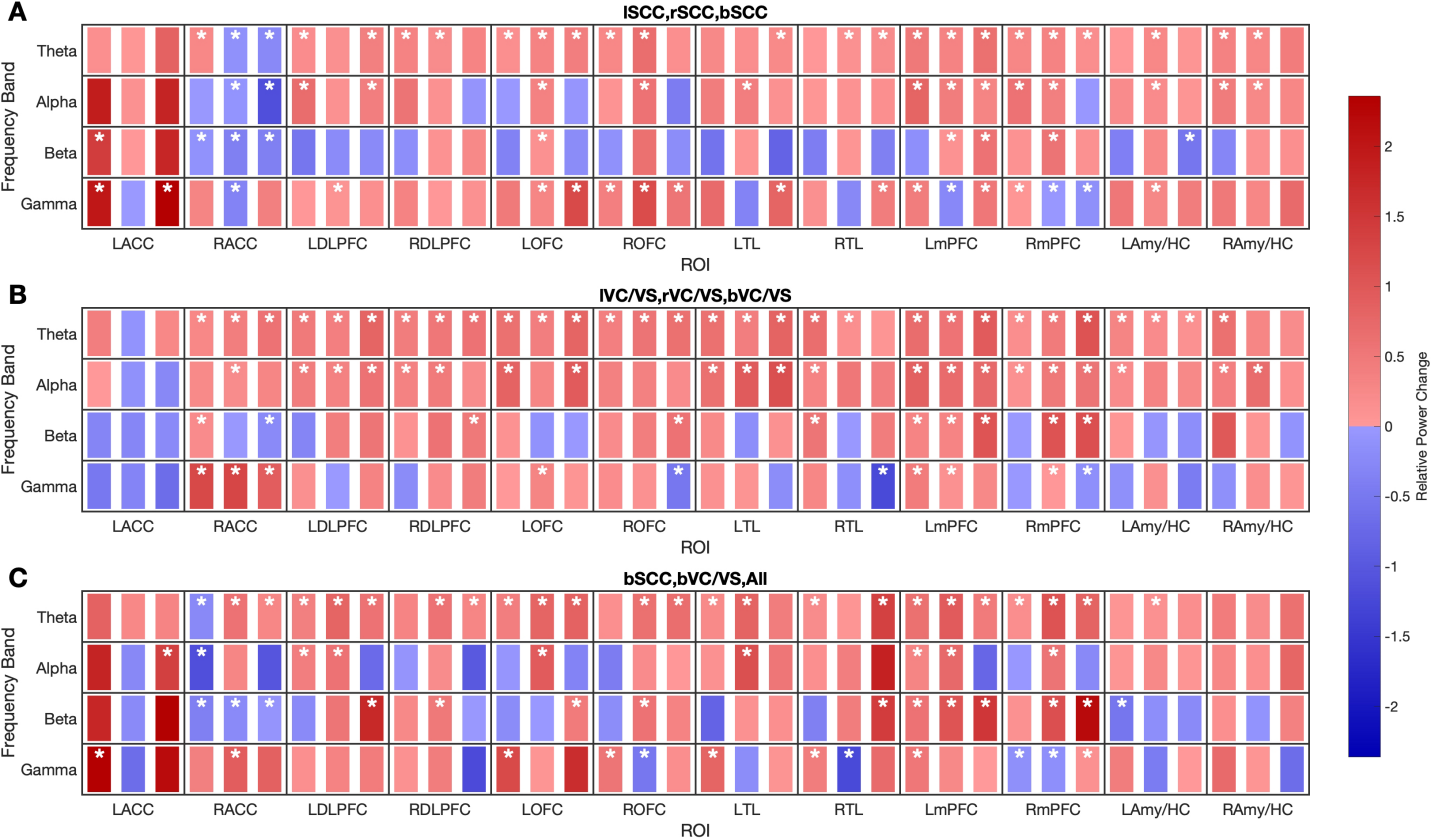
Heatmap of estimated relative power changes across three subjects for each frequency band (y-axis) and ROI (x-axis) under SCC **(A)**, VC/VS **(B)**, and All (bilateral SCC and VC/VS) **(C)** stimulation. Each cell is divided into three sub-rectangles representing left unilateral stimulation, right unilateral stimulation, and bilateral stimulation for single-target stimulations **(A, B)** and SCC, VC/VS, and bilateral stimulation of SCC and VC/VS for multi-target stimulation **(C)**. Within each cell, three colored sub- rectangles indicate the modulation pattern for each stimulation condition, ROI, and frequency band: red denotes increased activity, blue denotes decreased activity, and asterisks (*) denote statistically significant changes.

**Figure 2** illustrates the brain regions and frequency bands modulated by unilateral and bilateral SCC, VC/VS stimulation (**Figures 2A, B**), as well as multi-target stimulation (simultaneous bilateral SCC and VC/VS; **Figure 2C**). Across conditions, significant LFP power modulation was predominantly characterized by increases in theta band power across multiple brain regions.

Several exceptions to this pattern were observed. Theta and alpha band suppression occurred in right ACC in response to right SCC and bilateral SCC stimulation. Gamma band suppression was observed in right mPFC, right OFC, and right TL in response to bilateral VC/VS stimulation; in right mPFC in response to bilateral SCC stimulation; and in bilateral mPFC and right ACC during right SCC stimulation. Beta band suppression occurred in left Amy/HC during bilateral SCC stimulation and in right ACC during unilateral and bilateral SCC, bilateral VC/VS, and multi-target stimulation.

Notably, many regions modulated by bilateral stimulation overlapped with those independently modulated by each unilateral target. For example, right VC/VS, left VC/VS, and bilateral VC/VS stimulation all modulated bilateral DLPFC. In a similar manner, multi-target stimulation modulated power in regions also subject to modulation by bilateral single-target SCC and VC/VS stimulations alone. Left OFC and left DLPFC, for instance, were modulated by bilateral SCC, bilateral VC/VS, and multi-target (SCC + VC/VS) stimulation.

The pooled LME includes a random intercept for subject nested in dataset and trial (1| subject: dataset: trial) to quantify the subject variability. The random effect standard deviation for subject: dataset: trial (0.232) and channel (0.561) were small relative to the residual standard deviation (3.18), with subject: dataset: trial accounting for only 0.5% of the total variance. This indicates that the between-subject and between-channel variability is small and thus the effects are consistent across individuals.

### 3.2 Interaction in multi-target and bilateral SCC and VC/VS stimulation

**Figure 3:**
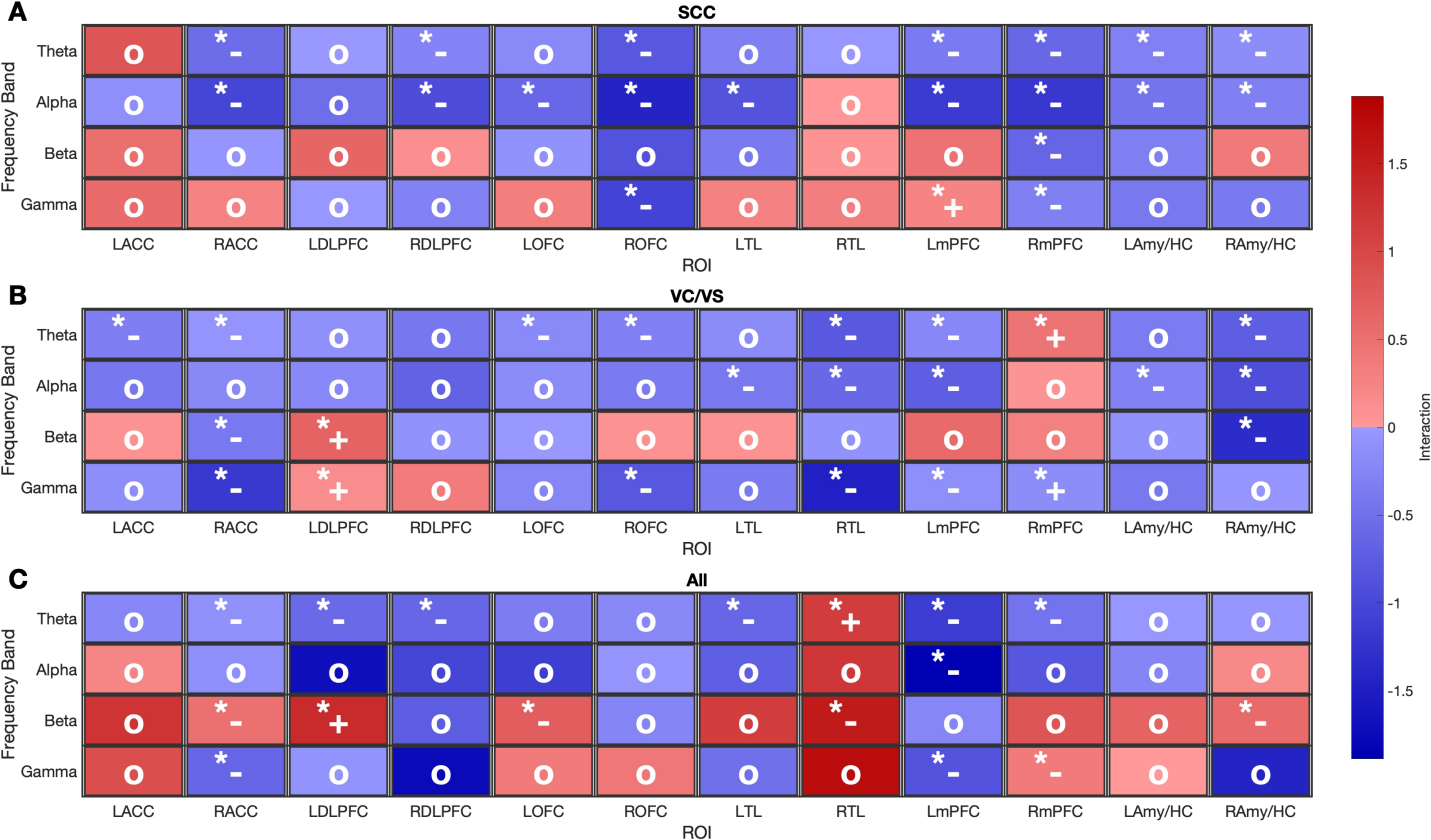
Heatmaps display the median interaction values (Δ*P_AB_ −* (Δ*P_A_* + Δ*P_B_*)) for each stimulation combination (**(A)** bilateral SCC, **(B)** bilateral VC/VS, **(C)** simultaneous bilateral SCC and VC/VS) and for each ROI (x-axis) and frequency band (y-axis). Significance levels (*) are shown at the top left corner of each cell and super-additive, sub-additive, and additive interactions are represented by +*, −*, and o.

Comparison of depression network modulation patterns between unilateral versus bilateral and single vs. multi target stimulation (**Figure 3**) reveals that interactions are dominated by additive or sub-additive effects, with super-additive effects occurring only rarely.

For single-target stimulation (**Figures 3A, B**), super-additive interactions were primarily observed at higher frequencies such as gamma band power with bilateral SCC stimulation in left mPFC and with bilateral VC/VS stimulation in left DLPFC and right mPFC. In contrast, during multi-target stimulation (**Figure 3C**), super-additive interactions occurred across both lower and higher frequencies, including the beta band in left DLPFC and at the theta band in right TL.

The pooled LME model includes a random intercept for subject nested in dataset and trial (1| subject: dataset: trial) to quantify the subject variability. The random effect standard deviation for subject: dataset: trial (0.289) and channel (0.624) were small relative to the residual standard deviation (2.484), with subject: dataset: trial accounting for only 1.3% of the total variance. This indicates that the between-subject and between-channel variability is small and thus the effects are consistent across individuals.

### 3.3 Additive interaction model

**Figure 4:**
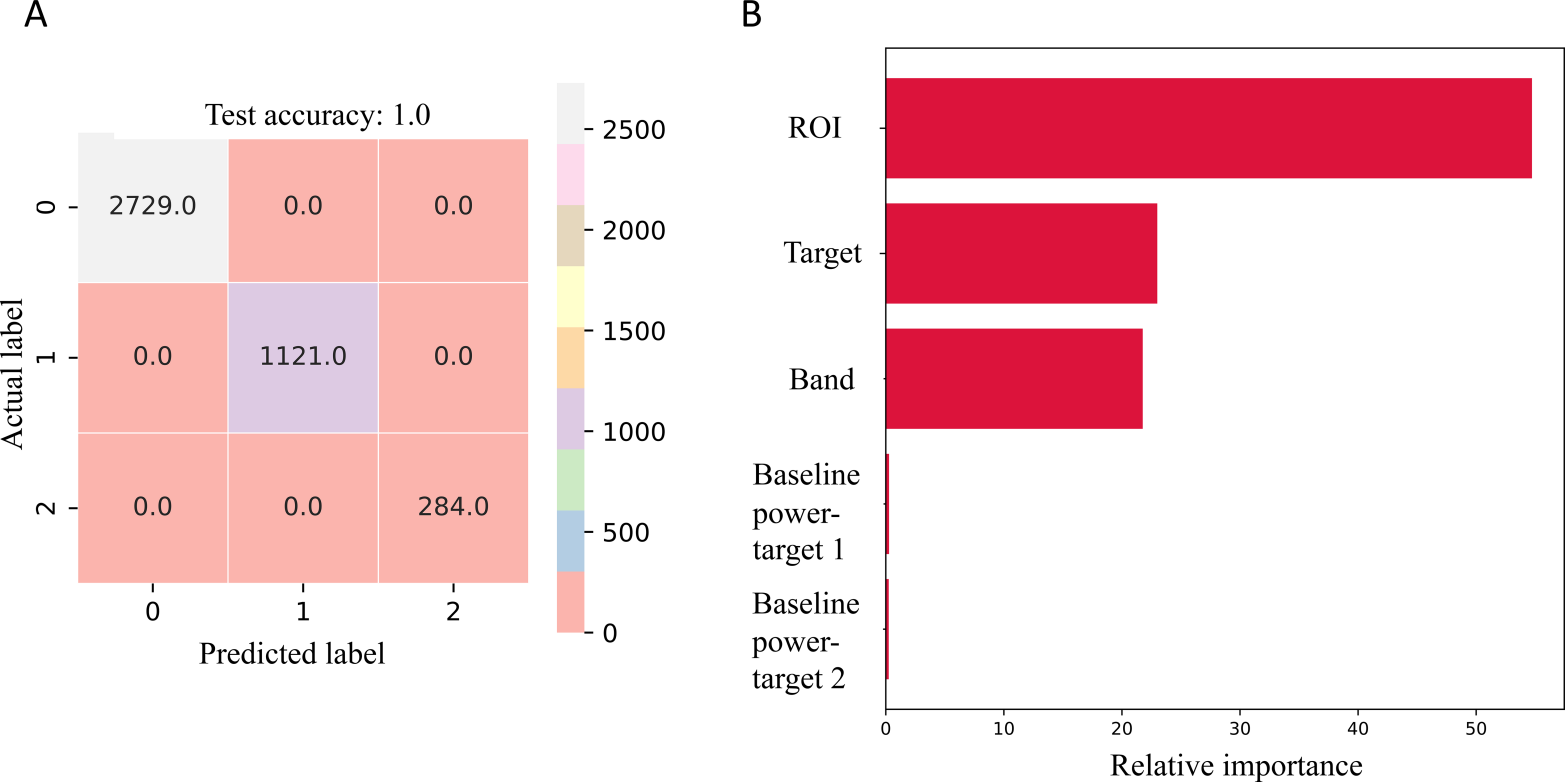
**(A)** Confusion matrix of the test data showing the classification performance of decision tree model across the three additive interaction classes of multi-lead stimulation. **(B)** Feature importance rankings derived from the decision tree model, highlighting the most important variables contributing to the classification of the additive interaction classes of multi-target stimulation.

The decision tree machine learning algorithm used to model the interaction effects of combining stimulation across hemispheres and targets, demonstrated a high predictive accuracy. The decision tree classifier achieved a train and test accuracy of 100%. and all additive classes had an F1-score of 1, as shown in the confusion matrix in **Figure 4** and **Table 1** and **Table 2**.

**Table 1:** Summary of decision tree classifier results for the test subset.

Class	Precision	Recall	F1-score	Support
Additive	1	1	1	2729
Sub-additive	1	1	1	1121
Super-additive	1	1	1	284

**Table 2:** Summary of decision tree classifier results for the train subset.

Class	Precision	Recall	F1-score	Support
Additive	1	1	1	6362
Sub-additive	1	1	1	2633
Super-additive	1	1	1	651

Feature importance analysis identified ROI, stimulation target, and frequency band as the most influential predictors, contributing 54.7%, 23%, and 21.8%, to the overall model decisions, respectively, while the pre-stimulation power of ROI during stimulation with target 1 and target 2 contributed 0.3% and 0.2% to the overall model decision. The resultant decision tree (**Supplementary Figure 1**) highlights the most influential pathways in the decision-making process.

## 4 Discussion

The goal of DBS regardless of therapeutic target or symptom is to modulate distributed brain networks, rather than simply induce local changes in neurophysiological activity Mu¨ller et al. (24)Drysdale et al. (25). In patients with Parkinson’s disease, for example, subthalamic nucleus stimulation modulates motor networks by reducing beta band cortical synchronization, particularly over sensorimotor and prefrontal areas Weiss et al. (26). Similarly, Riva-Posse et al. Riva-Posse et al. (9) used tractography to demonstrate that SCC DBS modulates mPFC as well as the rostral and dorsal ACC via specific white matter tracts. Importantly, these connectivity patterns, rather than the anatomical location of the electrode contacts, distinguished responders from non-responders.

While the effects of neuromodulation with single-target stimulation is easiest to study, DBS for TRD almost always involves bilateral stimulation. Yet the neuromodulatory effects and resulting interactions of bilateral stimulation remain relatively understudied and incompletely understood. Greater insight into these interactions are particularly valuable as multisite stimulation paradigms, including multiple targets, such as VC/VS and SCC, are considered as therapeutic options, given both overlapping and distinct connectivity patterns of these regions Lozano et al. (8) Makris et al. (13).

Our analyses revealed several key findings regarding power modulation by SCC and VC/VS stimulation. This study builds on our prior subject-level analysis in two individuals Allawala et al. (27) by incorporating data from three subjects, enabling group-level inference and assessment of additive interaction types in relative power change associated with bilateral and multi-target stimulation.

Examining the individual effects of unilateral and bilateral stimulation revealed that both targets can induce significant power modulation across different ROIs and frequency bands which can be informative in the selection of stimulation target. For example, **Figure 2** shows modulation of left ACC in response to left SCC stimulation (in beta and gamma bands) but not right SCC stimulation. Given the inverse relationship between depression severity and high frequency ACC power Mayberg (28) Xiao et al. (29) Kabotyanski et al. (30), this left ACC modulation may explain why left SCC stimulation contributes more to therapeutic efficacy in SCC DBS for TRD than does right SCC stimulation Conroy et al. (31).

Importantly, assessing the combined effects of both stimulation targets allowed us to evaluate interaction types, providing a nuanced understanding of synergy and antagonism. Super-additive interactions, where the combined effect exceeded the sum of individual effects, indicating reinforcement, were observed in the gamma band in regions such as left mPFC (bilateral SCC stimulation) and right mPFC and left DLPFC (bilateral VC/VS stimulation). Conversely, sub-additive interactions, where the combined effect was smaller than expected, occurred in both theta and alpha bands across several regions: right ACC, right DLPFC, right OFC, bilateral mPFC, bilateral Amy/HC (bilateral SCC stimulation) and right TL, left mPFC, right Amy/HC (bilateral VC/VS stimulation). Additive interactions, where the confidence interval included zero indicating no statistically significant deviation from expected additive effects, were observed across all frequency bands in left ACC, right OFC, left Amy/HC in response to simultaneous bilateral SCC and VC/VS stimulation. We note that with small sample size non-significant effects (additive interactions) should be interpreted with caution. To allow the readers to evaluate the magnitude and uncertainly of each additive interaction, we have reported the bootstrap median interaction and 95% confidence intervals for all ROI-frequency band combinations that were presented as additive in **Figure 3** in the **Supplementary Table 2**.

As previously reported Xiao et al. (29) Kabotyanski et al. (30), increased high-frequency and decreased low-frequency activity in prefrontal regions is associated with improved depression severity. Similarly, Clark et al. Clark et al. (32) reported an inverse correlation between beta power in subgenual ACC and depression severity. Our results (**Figure 3**) show that super-additive interactions in higher frequency bands (gamma and beta) occur in left mPFC with bilateral SCC stimulation, in left DLPFC with bilateral VC/VS stimulation, and in left DLPFC with multi-target stimulation. These super-additive interactions suggest increased stimulation efficiency may be achievable when the therapeutic goal is to enhance high- frequency power in these ROIs. At lower frequency bands, however, interactions were predominantly additive or sub-additive across most stimulation targets and ROIs assessed, with exceptions in right mPFC (bilateral VC/VS stimulation) and right TL (multi-target stimulation). This pattern suggests that synergistic effects may not extend uniformly across all frequency bands. Future studies should correlate the LFP power interactions with clinical and behavioral outcomes to strengthen the translational relevance of these interactions.

While our study provides a detailed map of stimulation interactions across ROIs and frequency bands, the limited number of subjects constrains the generalizability of these findings. Nevertheless, these analyses offer a robust framework for predicting additive and non-additive interactions and may guide future studies on combinatorial stimulation paradigms affect neuromodulation outcomes.

## Data Availability

The raw data supporting the conclusions of this article will be made available by the authors, without undue reservation.
