# Supplementary material for: Combinatorial effects of multi-site stimulation on depression-related brain regions: clinical data analysis and predictive modeling
Source: Front Psychiatry. 2026 Apr 30;17:1808486. doi: 10.3389/fpsyt.2026.1808486 (PMC13171579; doi:10.3389/fpsyt.2026.1808486)
Supplement: Supplementary file 2 [file Table1.docx]

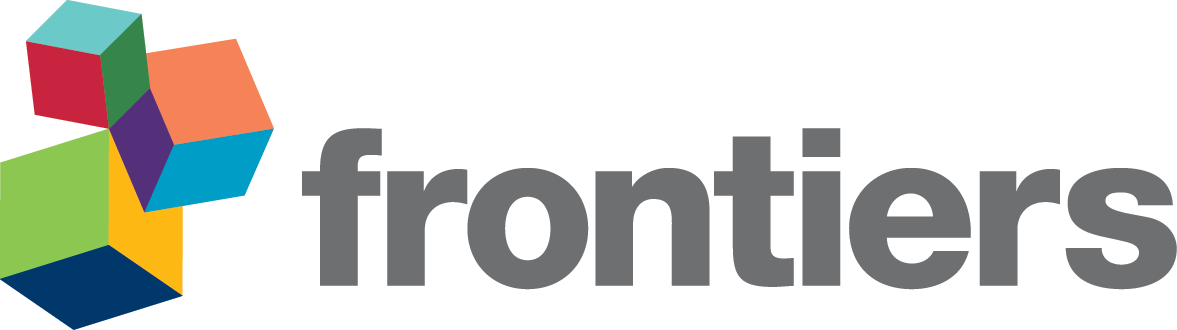


Supplementary Material

1. **SUPPLEMENTARY TABLES AND FIGURES**

# Tables

**Table S1.** Deep Brain Stimulation parameters. I: current amplitude, PW: Pulse Width, Freq.: Frequency, IPhD: Interphase delay, IPuD: Interpulse delay, Ratio: ratio of the first-phase amplitude to the second-phase amplitude, Day: day post-implantation for the recording session, Bhvrl: Behavioral, rVC/VS: right ventral capsule/ventral striatum, lVC/VS: left ventral capsule/ventral striatum, rSCC: right subcallosal cingulate, lSCC: left subcallosal cingulate, S1: Subject1, S2: Subject2, S3: Subject3.

| **Subject** | **Target** | **Contact** | **I, PW, Freq, IPhD** | **IPuD** | **Polarization, Ratio** | **Guidance** | **Day** |
| --- | --- | --- | --- | --- | --- | --- | --- |
| S1 | rSCC | 5,6,7 | 1.6mA,100*µ***s**,130Hz,100*µ***s** | 8ms | anodal-first,8 | — | 6 |
| S1 | lSCC | 5,6,7 | 1.6mA,100*µ***s**,130Hz,100*µ***s** | 8ms | anodal-first,8 | — | 6 |
| S1 | rVC/VS | 2,5 | 2.4mA,180*µ***s**,130Hz,100*µ***s** | 8ms | anodal-first,8 | — | 6 |
| S1 | lVC/VS | 2,5 | 2.4mA,180*µ***s**, 130Hz,100*µ***s** | 8ms | anodal-first,8 | — | 6 |
| S2 | rSCC | 6,8 | 2.1,0.2mA,100*µ***s**, 130Hz,100*µ***s** | 9ms | cathodal-first,1 | Illumina | 6 |
| S2 | lSCC | 2,3,6 | 0.3,2.8,0.9mA,100*µ***s**, 130Hz,100*µ***s** | 9ms | cathodal-first,1 | Illumina | 6 |
| S2 | rVC/VS | 2,3 | 9.8, 9.6mA,100*µ***s**, 130Hz,100*µ***s** | 9ms | cathodal-first,1 | Illumina | 6 |
| S2 | lVC/VS | 2,3 | 3.6, 6.7mA,180*µ***s**, 130Hz,100*µ***s** | 9ms | cathodal-first,1 | Illumina | 6 |
| S2 | rSCC | 2,5 | 3,3mA,90*µ***s**, 130Hz,100*µ***s** | 9ms | cathodal-first,1 | Bhvrl | 8 |
| S2 | lSCC | 8 | 6mA,90*µ***s**, 130Hz,100*µ***s** | 9ms | cathodal-first,1 | Bhvrl | 8 |
| S2 | rVCVS | 2 | 5mA,90*µ***s**, 130Hz,100*µ***s** | 9ms | cathodal-first,1 | Bhvrl | 8 |
| S2 | lVC/VS | 5 | 5mA,90*µ***s**, 130Hz,100*µ***s** | 9ms | cathodal-first,1 | Bhvrl | 8 |
| S3 | rSCC | 5,6,7 | 1.7mA,180*µ***s**, 130Hz,100*µ***s** | 9ms | cathodal-first,1 | Image | 6 |
| S3 | lSCC | 1 | 5mA,180*µ***s**, 130Hz,100*µ***s** | 9ms | cathodal-first,1 | Image | 6 |
| S3 | rVC/VS | 4 | 5mA,180*µ***s**, 130Hz,100*µ***s** | 9ms | cathodal-first,1 | Image | 6 |
| S3 | lVC/VS | 4 | 5mA,180*µ***s**, 130Hz,100*µ***s** | 9ms | cathodal-first,1 | Image | 6 |
| S3 | rSCC | 5,6,7 | 2mA,90*µ***s**, 130Hz,100*µ***s** | 9ms | cathodal-first,1 | Bhvrl | 9 |
| S3 | lSCC | 1 | 6mA,90*µ***s**, 130Hz,100*µ***s** | 9ms | cathodal-first,1 | Bhvrl | 9 |
| S3 | rVC/VS | 2 | 6mA,90*µ***s**, 130Hz,100*µ***s** | 9ms | cathodal-first,1 | Bhvrl | 9 |
| S3 | lVC/VS | 6 | 6mA,90*µ***s**, 130Hz,100*µ***s** | 9ms | cathodal-first,1 | Bhvrl | 9 |

Table S2: Summary of additive interaction classifications across stimulation targets, ROIs, and frequency bands. Results are reported as median Interaction with 95% bootstrap confidence intervals (CI) and FDR-corrected *p*-values.

| **Stim** | **ROI** | **Band** | **Median** | **CI High** | **CI Low** | *p***-value (FDR)** |
| --- | --- | --- | --- | --- | --- | --- |
| SCC | LACC | Theta | 0.8358 | 1.8345 | -1.1507 | 0.6305 |
| SCC | LACC | Alpha | -0.1187 | 0.3409 | -0.5946 | 0.8293 |
| SCC | LACC | Beta | 0.5124 | 1.1397 | -0.2073 | 0.6305 |
| SCC | LACC | Gamma | 0.5554 | 1.1243 | -0.0774 | 0.4087 |
| SCC | LAmy/HC | Beta | -0.3144 | 0.0934 | -1.1953 | 0.4087 |
| SCC | LAmy/HC | Gamma | -0.4004 | 0.3484 | -1.2468 | 0.6265 |
| SCC | LDLPFC | Theta | -0.0170 | 0.1450 | -0.2476 | 0.8520 |
| SCC | LDLPFC | Alpha | -0.5347 | 0.6226 | -0.7810 | 0.1833 |
| SCC | LDLPFC | Beta | 0.6281 | 1.0099 | -0.2824 | 0.4773 |
| SCC | LDLPFC | Gamma | -0.0294 | 0.0602 | -0.0864 | 0.4087 |
| SCC | LOFC | Theta | -0.1939 | 0.1388 | -0.6353 | 0.4087 |

*Continued on next page*

***Supplementary Material***

Table S2 – *continued*

| **Stim** | **ROI** | **Band** | **Median** | **CI High** | **CI Low** | *p***-value (FDR)** |
| --- | --- | --- | --- | --- | --- | --- |
| SCC | LOFC | Beta | -0.0929 | 0.5450 | -1.5882 | 0.6305 |
| SCC | LOFC | Gamma | 0.3388 | 1.3389 | -0.8867 | 0.6305 |
| SCC | LTL | Theta | -0.3124 | 0.3826 | -0.7836 | 0.4087 |
| SCC | LTL | Beta | -0.3882 | 0.0908 | -1.2284 | 0.4087 |
| SCC | LTL | Gamma | 0.2890 | 1.0147 | -0.0573 | 0.4087 |
| SCC | LmPFC | Beta | 0.4785 | 0.6370 | -0.0526 | 0.1996 |
| SCC | RACC | Beta | -0.0451 | 0.0066 | -0.0803 | 0.1996 |
| SCC | RACC | Gamma | 0.2690 | 0.2875 | -0.1764 | 0.1996 |
| SCC | RAmy/HC | Beta | 0.3828 | 2.5515 | -0.2158 | 0.3840 |
| SCC | RAmy/HC | Gamma | -0.3897 | 0.6330 | -0.4656 | 0.6265 |
| SCC | RDLPFC | Beta | 0.1216 | 1.0641 | -0.8456 | 0.8293 |
| SCC | RDLPFC | Gamma | -0.2886 | 0.3761 | -0.5805 | 0.6305 |
| SCC | ROFC | Beta | -0.8789 | 0.0112 | -1.2860 | 0.3840 |
| SCC | RTL | Theta | -0.0287 | 0.0504 | -0.3004 | 0.4087 |
| SCC | RTL | Alpha | 0.0244 | 0.0917 | -1.6935 | 0.8293 |
| SCC | RTL | Beta | 0.0606 | 0.2107 | -0.4781 | 0.6305 |
| SCC | RTL | Gamma | 0.3266 | 0.5440 | -0.0967 | 0.1554 |
| VC/VS | LACC | Alpha | -0.4236 | 0.2526 | -0.6057 | 0.4224 |
| VC/VS | LACC | Beta | 0.0787 | 0.1744 | -0.2284 | 0.6768 |
| VC/VS | LACC | Gamma | -0.1334 | 0.0831 | -0.2151 | 0.1421 |
| VC/VS | LAmy/HC | Theta | -0.3597 | 0.0403 | -0.5180 | 0.1477 |
| VC/VS | LAmy/HC | Beta | -0.0854 | 0.8423 | -0.9014 | 0.6768 |
| VC/VS | LAmy/HC | Gamma | -0.3953 | 0.6249 | -0.7894 | 0.4230 |
| VC/VS | LDLPFC | Theta | -0.1172 | 0.2360 | -0.3520 | 0.6400 |
| VC/VS | LDLPFC | Alpha | -0.2127 | 1.1149 | -1.1684 | 0.7304 |
| VC/VS | LOFC | Alpha | -0.1639 | 0.2863 | -1.2158 | 0.4224 |
| VC/VS | LOFC | Beta | -0.0149 | 0.5195 | -0.5649 | 0.7480 |
| VC/VS | LOFC | Gamma | -0.2358 | 0.4984 | -1.1465 | 0.4230 |
| VC/VS | LTL | Theta | -0.1666 | 0.1207 | -0.7153 | 0.4224 |
| VC/VS | LTL | Beta | 0.0776 | 0.2232 | -0.2729 | 0.5591 |
| VC/VS | LTL | Gamma | -0.3727 | 0.3611 | -0.7340 | 0.4224 |
| VC/VS | LmPFC | Beta | 0.5731 | 0.7170 | -0.5669 | 0.7304 |
| VC/VS | RACC | Alpha | -0.2195 | 0.1463 | -0.3864 | 0.6768 |
| VC/VS | RAmy/HC | Gamma | -0.0521 | 1.2317 | -1.6699 | 0.7480 |
| VC/VS | RDLPFC | Theta | -0.4136 | 0.0558 | -0.6468 | 0.1421 |
| VC/VS | RDLPFC | Alpha | -0.6774 | 0.3840 | -1.0308 | 0.4975 |
| VC/VS | RDLPFC | Beta | -0.0918 | 1.3484 | -1.1763 | 0.6768 |
| VC/VS | RDLPFC | Gamma | 0.3596 | 1.2138 | -0.8142 | 0.7024 |
| VC/VS | ROFC | Alpha | -0.3738 | 0.1054 | -0.6961 | 0.1421 |
| VC/VS | ROFC | Beta | 0.0785 | 1.0075 | -0.3825 | 0.6391 |
| VC/VS | RTL | Beta | -0.0939 | 0.1788 | -1.5152 | 0.7480 |

*Continued on next page*

***Supplementary Material***

Table S2 – *continued*

| **Stim** | **ROI** | **Band** | **Median** | **CI High** | **CI Low** | *p***-value (FDR)** |
| --- | --- | --- | --- | --- | --- | --- |
| VC/VS | RmPFC | Alpha | 0.0681 | 0.5308 | -0.0860 | 0.7304 |
| VC/VS | RmPFC | Beta | 0.2962 | 0.3759 | -1.5827 | 0.7304 |
| All | LACC | Theta | -0.2250 | 0.3153 | -0.4173 | 0.6400 |
| All | LACC | Alpha | 0.2377 | 1.0089 | -0.0562 | 0.5176 |
| All | LACC | Beta | 1.2395 | 2.4072 | -0.0057 | 0.4301 |
| All | LACC | Gamma | 0.9285 | 2.0615 | -0.1969 | 0.6541 |
| All | LAmy/HC | Theta | -0.0261 | 0.1053 | -0.2204 | 0.6400 |
| All | LAmy/HC | Alpha | -0.2458 | 0.1069 | -0.3238 | 0.1837 |
| All | LAmy/HC | Beta | 0.6638 | 0.7697 | -0.1841 | 0.1837 |
| All | LAmy/HC | Gamma | 0.0069 | 0.2064 | -0.1508 | 0.8807 |
| All | LDLPFC | Alpha | -1.7037 | 0.4289 | -2.3386 | 0.1837 |
| All | LDLPFC | Gamma | -0.0794 | 0.2715 | -0.2079 | 0.5176 |
| All | LOFC | Theta | -0.3488 | 0.2201 | -0.5547 | 0.1837 |
| All | LOFC | Alpha | -1.1146 | 0.7090 | -1.3753 | 0.1837 |
| All | LOFC | Gamma | 0.3701 | 0.6164 | -0.7711 | 0.6400 |
| All | LTL | Alpha | -0.7120 | 0.0152 | -1.8251 | 0.1547 |
| All | LTL | Beta | 1.1213 | 1.5637 | -0.5554 | 0.6400 |
| All | LTL | Gamma | -0.5107 | 0.0602 | -2.8732 | 0.6400 |
| All | LmPFC | Beta | -0.2345 | 0.0524 | -1.2508 | 0.5176 |
| All | RACC | Alpha | -0.1188 | 0.8467 | -0.3008 | 0.7685 |
| All | RAmy/HC | Theta | -0.0387 | 2.6672 | -0.4641 | 0.6698 |
| All | RAmy/HC | Alpha | 0.2048 | 2.5010 | -0.6823 | 0.6400 |
| All | RAmy/HC | Gamma | -1.4447 | 3.2406 | -2.1263 | 0.6400 |
| All | RDLPFC | Alpha | -1.0304 | 0.0494 | -1.6727 | 0.4301 |
| All | RDLPFC | Beta | -0.7397 | 0.8517 | -1.3197 | 0.8900 |
| All | RDLPFC | Gamma | -1.7647 | 0.5159 | -2.1683 | 0.5176 |
| All | ROFC | Theta | -0.2141 | 0.9027 | -0.3812 | 0.6400 |
| All | ROFC | Alpha | -0.0714 | 0.9360 | -0.5230 | 0.8885 |
| All | ROFC | Beta | -0.2579 | 0.6785 | -0.4859 | 0.1547 |
| All | ROFC | Gamma | 0.4234 | 1.8620 | -0.7250 | 0.6400 |
| All | RTL | Alpha | 1.2162 | 1.3613 | -1.1058 | 0.6400 |
| All | RTL | Gamma | 1.7393 | 1.9692 | -0.3875 | 0.6400 |
| All | RmPFC | Alpha | -0.8240 | 0.0665 | -1.0848 | 0.5176 |
| All | RmPFC | Beta | 0.8441 | 1.1302 | -0.0069 | 0.5176 |

# Figures

***Supplementary Material***

Figure S1: Decision tree illustrating classification of additive interaction. Each internal node represents a binary split, leading eventually to terminal (leaf) nodes that display group classification (0 = additive, 1 = sub-additive, 2 = super-additive) and their associated counts. The hierarchy of the nodes conveys the relative importance of variables in the classification process. The node labeled Areapprime corresponds to the ROI, Stimprime to stimulation target, bandprime to frequency band, y1preinitial to pre-stimulation power of ROI during stimulation with target 1, and y2preinitial to pre-stimulation power of ROI during stimulation with target 2. Using this decision tree and knowing the features, one can determine the interaction class by following an appropriate path in this tree.
