# Supplementary material for: Combinatorial effects of multi-site stimulation on depression-related brain regions: clinical data analysis and predictive modeling
Source: Front Psychiatry. 2026 Apr 30;17:1808486. doi: 10.3389/fpsyt.2026.1808486 (PMC13171579; doi:10.3389/fpsyt.2026.1808486)
Supplement: Supplementary file 1 [file Image1.pdf]

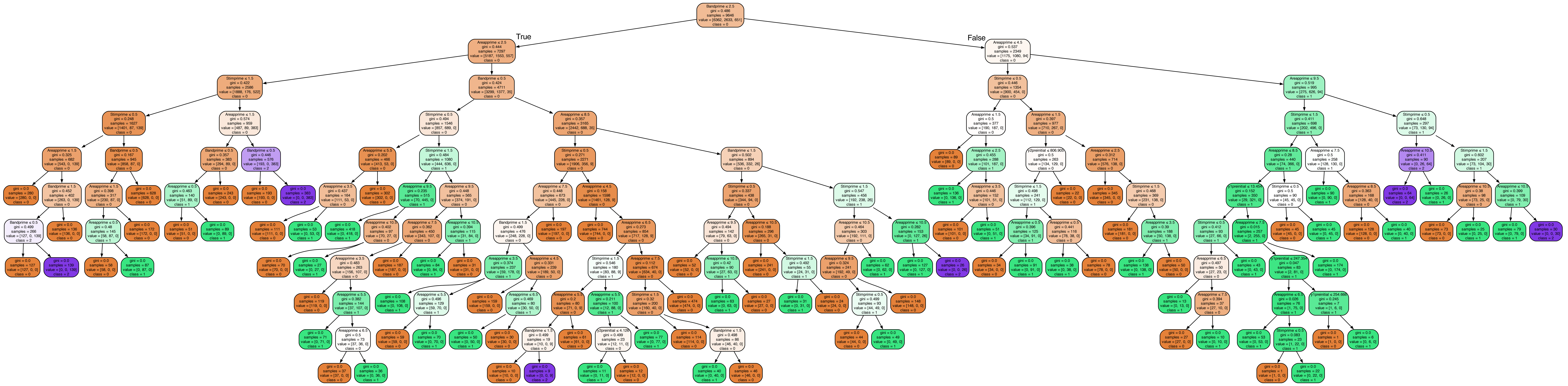
Bandprime ≤ 2.5
gini = 0.486
samples = 9646
value = [6362, 2633, 651]
class = 0
True
False
Areaprime ≤ 2.5
gini = 0.444
samples = 7297
value = [5187, 1553, 557]
class = 0
Areaprime ≤ 4.5
gini = 0.537
samples = 2349
value = [1175, 1080, 94]
class = 0
